# Suspect Screening of Polymer-Derived Additives in Accessible Water Compartments Nearby Brånåsen Landfill in Norway

**DOI:** 10.3390/molecules31111922

**Published:** 2026-06-02

**Authors:** Sara Abdelli, Luigi Mastrodonato, Vian Yasin, Niroshan Gajendra, Sissel Opsahl Viig, Davide Vione, Paola Calza, Laura Ferrando Climent

**Affiliations:** 1 Tracer Technology, Environmental Technology Section, Tracer Technology Department, Institute for Energy Technology, Instituttveien 18, 2007 Kjeller, Norway; sara.abdelli@ife.no (S.A.); vian.yasin@ife.no (V.Y.); niroshan.gajendra@ife.no (N.G.); sissel.opsahl.viig@ife.no (S.O.V.); 2 Dipartimento di Chimica, Università di Torino, Via Pietro Giuria 5, 10125 Torino, Italy; luigi.mastrodonato@upc.edu (L.M.); davide.vione@unito.it (D.V.); paola.calza@unito.it (P.C.)

**Keywords:** contaminants of emerging concern (CECs), polymer-derived additives, PFAS, aquatic ecosystems, landfill, screening, environmental risk

## Abstract

This study investigates the occurrence, distribution, and environmental implications of polymer-derived additives, including per- and polyfluoroalkyl substances (PFAS), in aquatic systems impacted by the Brånåsen legacy landfill in Norway. Water samples were collected from landfill leachate and from seven locations along the Nitelva River to evaluate both point-source contamination and downstream transport processes. UHPLC–HRMS and GC–MS/MS suspect screening analyses provided tentative evidence for dimethacrylate-related compounds, including features putatively assigned to DEGDMA and TEGDMA-related compounds, potentially associated with polymer degradation processes. Selected PFAS were instead quantified by targeted LC–MS/MS, with PFOS, PFOA, PFHxS, and PFHxA exhibiting the highest concentrations at the landfill tributary, reaching maximum concentrations of up to 780 ng L^−1^, and decreasing downstream consistently with dilution and hydrological mixing, while remaining detectable throughout the river reach. Temporal variation indicated short-term hydrological influences on contaminant mobility. The study highlights the role of legacy landfills as persistent sources of emerging pollutants in freshwater environments. These findings underscore the need for improved monitoring strategies and advanced treatment measures to address complex contaminant mixtures originating from historical waste disposal activities.

## 1. Introduction

Access to clean water is crucial for domestic consumption, agricultural activities, energy generation, industrial processes, and the sustainability of ecosystems. Nevertheless, the worldwide distribution of water stress and scarcity is significantly imbalanced, frequently associated with substantial environmental, health, and socio-economic challenges [1,2]. Population growth, urbanization, agriculture, and industrial activity are placing increasing pressure on water systems, driving demand for complex infrastructure to provide clean water and manage wastewater [3,4]. However, these same developments introduce a wide range of pollutants, including nutrients, heavy metals, toxic organic compounds, and pathogens, into aquatic environments from both point sources like industrial effluents and diffuse sources such as agricultural runoff. In many regions, inadequate wastewater treatment further contributes to contamination [5,6,7]. While sewerage systems have improved public health, they have also centralized pollutant discharge into waterways, shifting rather than eliminating environmental risks [8]. On the other hand, pollution patterns differ globally, for example, Latin America is dominated by organic pollution from sewered domestic wastewater, Africa by non-sewered waste, Asia by industrial effluents and Europe by agricultural and urban stormwater runoff [9,10]. In response, emerging water management strategies emphasize integrated, sustainable approaches that balance environmental protection with human and economic needs. Growing concern surrounds unregulated contaminants of emerging concern (CECs), which are often found in surface waters and point to the need for improved monitoring and treatment [3].

CECs are increasingly detected in aquatic environments due to advances in analytical technologies, yet they remain largely unregulated, posing growing ecological and regulatory challenges [11,12]. These CECs include pharmaceuticals, personal care products, industrial chemicals, and endocrine-disrupting compounds, all of which are recognized as significant emerging pollutants found across diverse environmental matrices. Although typically present at low concentrations, many of these substances are biologically active, persistent, and capable of bioaccumulation, raising serious environmental and human health concerns because conventional wastewater treatment technologies are not designed to effectively remove them [11,13]. In addition to these well-known classes of contaminants, increasing attention is being directed toward chemicals released from plastic materials through degradation, leaching, and the emission of additive compounds, which represent an emerging and insufficiently studied source of persistent pollutants in aquatic environments [14]. Plastics and related substances such as polymers and glue additives disposed of in landfills may contribute considerably to this issue, functioning as a major but under-investigated source of diverse chemical contaminants that enter waterways [15]. Landfill leachate is formed as water percolates through buried waste, and it mobilizes a wide array of polymer-associated compounds such as plasticizers, surfactants, UV stabilizers, cross-linkers, and monomer residues, many of which subsequently reach surrounding water bodies. Once discharged, these substances may persist in the environment, interact with aquatic organisms, or undergo partial degradation that generates secondary pollutants, thereby compounding ecological risks and complicating water quality management efforts.

CECs have become a central focus in environmental research, particularly in regions previously considered isolated from significant anthropogenic influence. Recent studies have demonstrated that subarctic and Arctic environments in Norway are not exempt from chemical pollution pressures [16,17,18,19]. Using FerryBox-equipped cargo ships and ferries, Brumovský et al. traced a suite of pharmaceuticals, artificial sweeteners, and other organic micropollutants from continental Europe to the Barents Sea, revealing their presence even in remote Norwegian marine waters [20]. These finding underscores both the limited removal efficiency of existing wastewater treatment plants (WWTPs) and the capacity of long-range atmospheric and oceanic transport mechanisms to disperse CECs over thousands of kilometers. The persistence of many CECs, driven by high polarity, low biodegradability, and limited photolysis, allows them to bypass conventional wastewater treatment and accumulate in aquatic environments. Pharmaceuticals like carbamazepine and markers such as sucralose consistently appear in Norwegian waters, indicating sustained human impact [20,21]. Their chronic low-level presence poses ecotoxicological risks, including endocrine disruption, behavioral changes, and shifts in microbial communities that may promote antibiotic-resistance gene dissemination [22].

Growing awareness of these risks has prompted substantial regulatory developments across Europe. Under the updated EU Landfill Directive, most recently amended in 2024, operators are now required to meet strengthened operational and technical standards, including robust leachate collection, monitoring, and treatment systems designed to prevent contamination of groundwater, surface waters, and soil. These rules reflect the EU’s broader transition toward a circular economy and include provisions for stricter controls on landfill operations, traceability of accepted waste, and mandatory engineered systems for leachate management. These requirements are reinforced by the European Environment Agency (EEA) [23], which highlights landfill leachate as a persistent environmental threat due to its complex mixture of pollutants, including pharmaceuticals and other emerging contaminants, while noting that existing wastewater treatment plants often lack the capacity to adequately treat leachate streams. This has driven intensified expectations for both monitoring frequency and advanced treatment technologies, particularly as data gaps remain in leachate characterization across Member States [24]. In parallel, the 2022 revision of the Urban Wastewater Treatment Directive (UWWTD) mandates quaternary treatment for priority CECs and introduces an Extended Producer Responsibility (EPR) scheme for the pharmaceutical and cosmetics sectors. Although Norway is not an EU Member State, its EEA participation means these evolving wastewater- and landfill-related requirements are likely to influence national policy, increasing pressure to modernize wastewater and leachate treatment, expand CEC monitoring, and align with upcoming EU obligations. This shift comes amid clear data gaps on CEC occurrence and behavior—especially in sparsely populated northern regions with limited advanced treatment—and broader EU policy signals (stricter landfill diversion, stronger monitoring, climate-accountability, and updated compliance frameworks) point toward more data-driven, treatment-intensive waste management in the coming years [25].

Within this broader context, the Brånåsen case study provides a concrete and nationally significant example of how legacy pollution and insufficient containment infrastructure can facilitate the release of diverse contaminants into the aquatic environment. Brånåsen, a former landfill in Viken County in the subarctic region of Skedsmo in Norway, has long been recognized as a potential point source of contamination due to inadequate leachate control and the absence of engineered barriers [26,27,28]. The landfill was closed in the 90s, and despite the high uncertainties on the composition of waste disposed, it is registered the discharge of industrial waste from chemicals, polymer and glue manufacturers. Unpublished municipal waste records and prior studies show potential contaminants in the area including PFAS and acrylamide compounds. There is a clear need to assess the occurrence of suspected pollutants through improved methods for identifying and characterizing sources of environmentally hazardous emissions in the Brånåsen and nearby area.

This study demonstrates source-tracking using chemical analyses of landfill leachate and upstream/downstream water samples from the Nitelva River. We combine suspect screening—useful for investigating compounds with known structures even when unconfirmed in a given environment—with targeted analyses of samples from the Brånåsen landfill area to assess the release of suspected polymer-derived additives and other inputs, and to characterize downstream transport and dilution in subarctic waters.

## 2. Results

### 2.1. Sampling Collection Parameters and Codification

The general parameters for the sample collection are shown in Table 1.

#### 2.1.1. Suspect Screening of Polymer-Derived Adhesive-like Substances

A suspect screening approach was performed to investigate the occurrence of acrylamide-type and related vinyl monomers in the analyzed samples. The suspect list included compounds, previously identified by the author work carried out in the area and substances characterized by α,β-unsaturated carbonyl structures commonly used as polymerizable intermediates in industrial formulations such as triethylene glycol dimethacrylate (TEGDMA) and diethylene glycol dimethacrylate (DEGDMA).

For retained candidates, identification confidence was evaluated according to the Schymanski framework using the evidence available for each analytical platform. For UHPLC–HRMS data, the experimental isotopic profile was compared with the theoretical isotopic distribution expected from the proposed elemental composition, and structural plausibility was further assessed by inspecting dd-MS^2^ spectra for diagnostic product ions, neutral losses, available mzCloud spectral-library matches, and chemically reasonable fragmentation pathways. Additional database searches were performed using ChemSpider and local suspect lists. For GC–MS/MS data, tentative assignments were supported by diagnostic fragmentation patterns and available spectral-library matches. Candidate compounds supported by sufficient spectral evidence, but lacking confirmation with authentic analytical standards, were assigned tentative confidence levels ranging from Schymanski Level 2 to Level 3, depending on the strength of the available evidence. Accordingly, DEGDMA and the main TEGDMA candidate were reported as tentative Schymanski Level 2 annotations when supported by diagnostic fragmentation and spectral-library matches, whereas the additional TEGDMA-related COMP286 features were conservatively reported as Schymanski Level 3 annotations because multiple structurally related or positional isomers could not be unambiguously distinguished. Therefore, all suspect-screening assignments reported in this study, whether obtained by UHPLC–HRMS or GC–MS/MS, should be regarded as tentative annotations rather than confirmed identifications, unless confirmed by comparison with authentic analytical standards.

**Figure 1:**
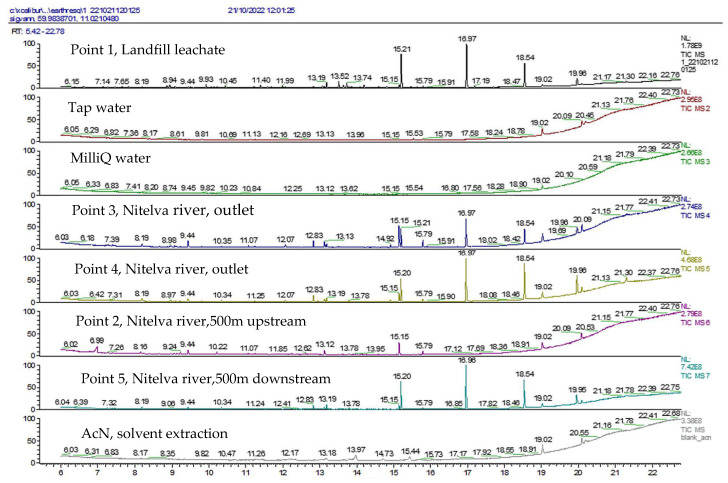
Total ion chromatograms (TICs) obtained by GC–MS/MS for the analyzed samples and controls. From top to bottom: Point 1 “landfill leachate”; tap water blank; Milli-Q water blank; Point 3 “Nitelva River outlet”; Point 4 “Nitelva River outlet”; Point 2 “Nitelva River 500 m upstream”; Point 5 “Nitelva River 500 m downstream”; and ACN solvent-extraction blank. TICs are vertically stacked and shown using relative intensity scaling to compare chromatographic patterns across samples.

Figure 1 shows the pattern along the samples taken downstream of the landfill discharge and at landfill leachate including the controls (MilliQ water, AcN used in the extractions, and tap water).

**Figure 2:**
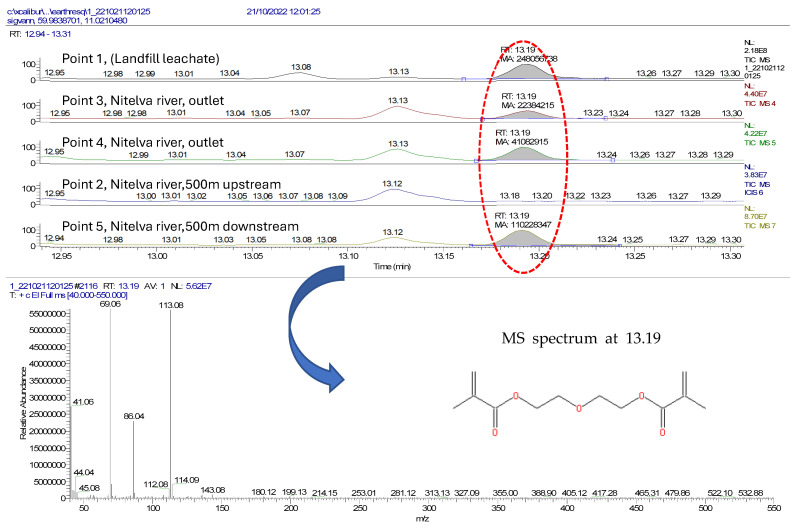
Tentative annotation of diethylenglycol dimethacrylate at point 1, 3, 4 and 5: Peak at 13.19 min with MS spectrum extracted from XIC at same time.

While examining the chromatographic data, a distinct signal was detected at 13.19 min after data processing, although it was not clearly distinguishable in the full-scale TIC shown in Figure 1. The signal was further examined using the extracted ion chromatogram (XIC) and MS spectrum, and the compound was tentatively annotated as diethylene glycol dimethacrylate, with a molecular mass of 242 and molecular formula C_12_H_18_O_5_ (90% match with the NIST library). This feature, including its retention time, detected sampling points, and nominal molecular mass, is summarized in Table 2. The tentative annotation of this component is presented in Figure 2 and  Figure S1 in the Supplementary Materials.

As reported by Alshali et al. [29], DEGDMA was identified as the principal leachable compound in some resin-based dental materials (e.g., SDR and x-tra base), accounting for more than 60% of the total chromatographic peak area in those formulations. This highlights its potential relevance as an environmental contaminant and underscores the need for further investigation and future targeted monitoring.

A second suspected component with high chromatographic peak intensity (10^8^) was detected at 15.21 min. After data processing, the peak was tentatively annotated as triethylene glycol dimethacrylate (TEGDMA), also referred to in some commercial sources as TGM3, with a molecular weight (MW) of 286 and a molecular formula of C_14_H_22_O_6_ (97% match with the NIST library). The tentative annotation of this component is presented in Table 2, Tables S2 and S3 (Supplementary Materials).

A third chromatographic feature was detected at 16.97 min and, for clarity, was assigned the designation COMP286a. This feature showed a nominal molecular ion at *m*/*z* 286 and an EI mass spectrum similar to that of triethylene glycol dimethacrylate (TEGDMA). However, in the absence of authentic standards or additional structural confirmation, this signal should be regarded as a TEGDMA-related isobaric feature rather than as a confirmed positional isomer. Two additional chromatographically resolved features, COMP286b and COMP286c, were detected at 18.54 and 19.96 min, respectively, showing similar mass spectral behavior, as also reported in the Supplementary Materials and summarized in Table 2.

Overall, the screening results suggest the tentative annotation of multiple dimethacrylate-related features in landfill-impacted samples, including diethylene glycol dimethacrylate (DEGDMA; MW 242) and several dimethacrylate-related MW 286 features tentatively related to triethylene glycol dimethacrylate (TEGDMA; hereafter COMP286). In general, mass spectra showed a strong agreement with the NIST library entries (90% match for DEGDMA and 97% for TEGDMA), providing added confidence to report these compounds as tentative (Schymanski Level 2) annotations in the absence of authentic standards.

Importantly, the MW 286 signal was not confined to a single chromatographic feature: in addition to the main peak at 15.21 min, several additional peaks with the same nominal mass and closely related spectral signatures were observed at distinct retention times (16.97, 18.54, and 19.96 min). These chromatographic features were therefore designated COMP286a, COMP286b, and COMP286c and reported as isobaric MW 286 candidates. Their similar fragmentation patterns suggest a relationship with dimethacrylate-type structures and indicate that putative TEGDMA-related structures may be proposed for these features. However, the available spectral information does not allow for unambiguous discrimination among possible structural alternatives, including possible isomeric forms, nor confirmation of the exact individual structures. Consequently, these features were conservatively assigned to Schymanski Level 3. The occurrence of multiple chromatographically resolved MW 286 features with similar spectral signatures suggests the possible presence of a complex mixture of structurally related dimethacrylate-type candidates in the environmental samples. These observations highlight the value of combining chromatographic separation, mass spectral information, library matching, and retention-time patterns when interpreting structurally related polymer-derived compounds in suspect-screening workflows.

#### 2.1.2. Suspect Screening of PFOA

**Figure 3:**
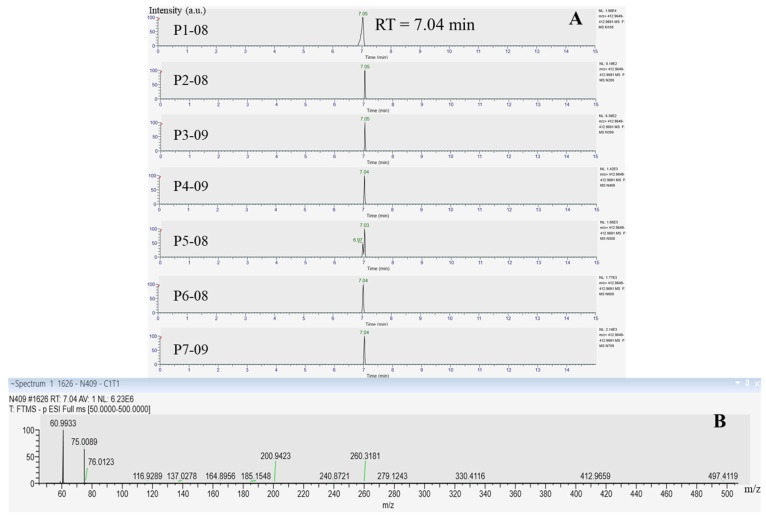
Confirmation of perfluorooctanoic acid (PFOA) by UHPLC–HRMS. (**A**) Extracted ion chromatograms showing detection of PFOA across representative water samples collected along the Nitelva River, with a consistent retention time of approximately 7.04 min. Sample identifiers correspond to sampling site (P1–P7) and sampling date. (**B**) MS/MS fragmentation spectrum obtained by data-dependent acquisition, displaying diagnostic fragment ions consistent with PFOA.

PFOA was selected as a representative model compound for the PFAS class and used for preliminary suspect-screening evaluation. It was consistently detected across the analyzed samples by UHPLC–HRMS using an Ultimate 3000 system coupled to a Q Exactive mass spectrometer (Thermo Scientific, GmbH, Bremen, Germany). Extracted ion chromatograms revealed a consistent chromatographic peak at approximately 7.04 min across multiple samples (Figure 3A). The assignment was supported by accurate mass measurements and by MS/MS spectra showing characteristic fragment ions consistent with the expected fragmentation pattern of PFOA (Figure 3B). In addition, comparison with an authentic analytical standard confirmed agreement in both retention time and mass-spectral features. Therefore, PFOA was assigned Schymanski Level 1 identification confidence within the suspect-screening workflow. Sample identifiers correspond to the sampling location (P1–P7) and the sampling date, where the number following the dash indicates the sampling day (e.g., P1–08 represents sampling point 1 collected on day 08). The detection of PFOA during suspect screening supported its inclusion in the subsequent targeted LC–MS/MS quantification, described in the following section.

#### 2.1.3. Quantification of PFAS

**Figure 4:**
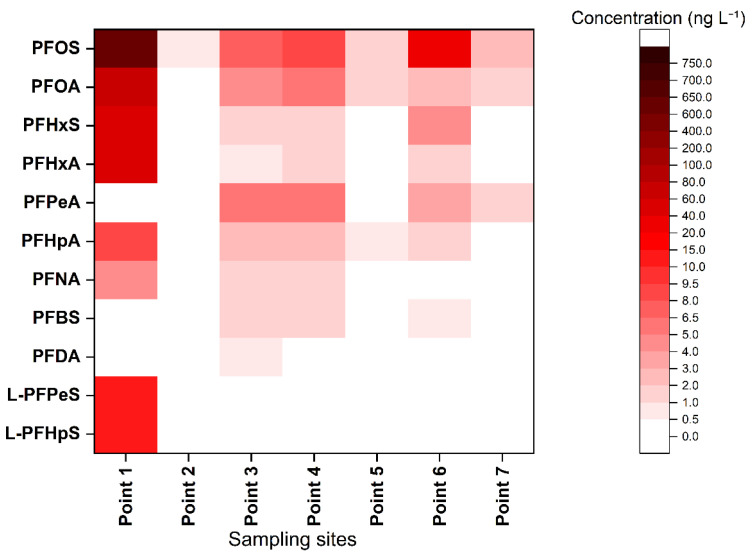
Spatial distribution of PFAS concentrations across the seven sampling sites along the Nitelva River. Colors represent mean concentrations (ng L^−1^) measured during the sampling campaign for individual PFAS compounds. Elevated concentrations were observed at Point 1, reflecting the influence of the landfill-associated tributary, while downstream sites exhibited progressively lower concentrations, indicating dilution and transport processes along the river.

**Figure 6:**
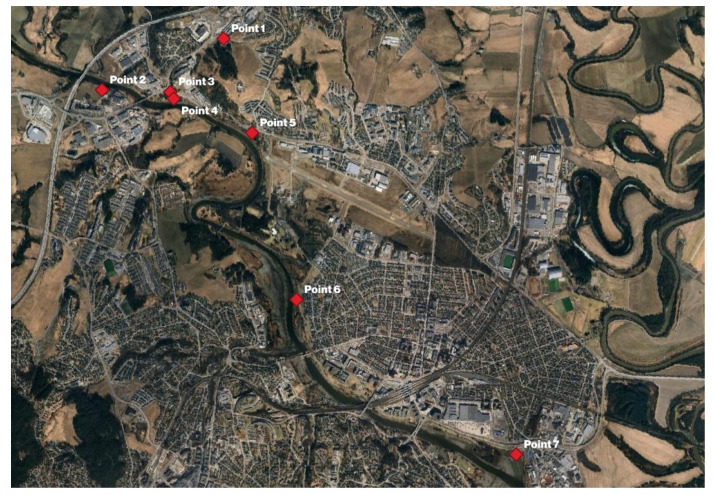
Spatial distribution of sampling points along the Nitelva River. Seven sites (Points 1–7) were strategically selected along the Nitelva River. The map was generated using QGIS (version 3.40.11) with satellite imagery provided via the QuickMapServices plugin.

PFAS concentrations revealed a pronounced spatial variability along the monitored section of the Nitelva River (Figure 4), clearly reflecting the influence of localized contamination sources and subsequent attenuation processes along the river system (see Figure 6 for a map of the sampling sites). PFOS was the dominant compound across most sampling sites and exhibited the highest concentrations among the quantified PFAS. The most elevated PFAS levels were observed at Point 1, where PFOS concentrations ranged from approximately 530 to 780 ng L^−1^ during the monitoring period (Figure 4). Elevated concentrations of PFOA, PFHxS, and PFHxA were also detected at this location, indicating a strong localized source input associated with landfill leachate. In contrast, Point 2 exhibited negligible PFAS concentrations across all sampling events, reflecting background river conditions prior to the influence of upstream contamination sources. Downstream of Point 1, PFAS concentrations decreased markedly within the main river channel. Points 3 and 4 showed moderate PFOS concentrations generally between approximately 5 and 10 ng L^−1^, while PFOA and PFHxS were detected at lower levels (Figure 4). Further downstream, Point 5 displayed relatively low PFAS concentrations overall, suggesting progressive dilution of contaminants along the river. Although PFAS levels generally declined downstream, measurable concentrations persisted throughout the river reach. Point 6 exhibited moderate PFOS and PFOA concentrations and showed notable variability between sampling events (Figure 4). Finally, Point 7 displayed relatively stable PFAS concentrations at low ng L^−1^ levels, reflecting the cumulative influence of upstream inputs following dilution and transport processes along the river. The observed downstream persistence of PFAS appears to be primarily controlled by diffuse urban inputs, particularly in proximity to populated areas, while the intrinsic recalcitrance of these compounds likely enhances their persistence during transport.

**Figure 5:**
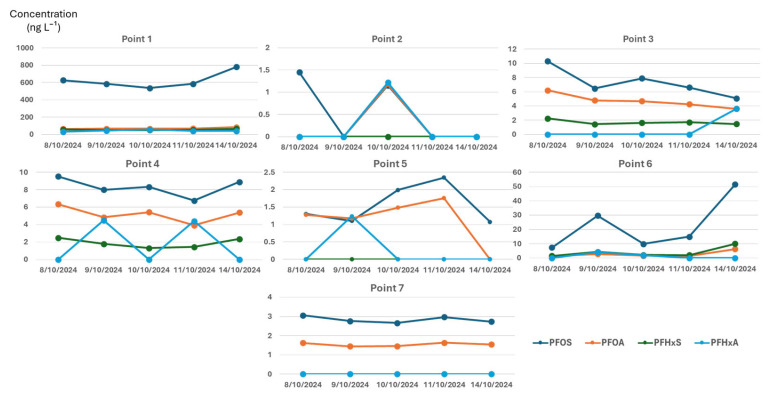
Temporal variation of selected PFAS concentrations (PFOS, PFOA, PFHxS, and PFHxA) across the seven sampling sites during the October monitoring campaign. Line plots illustrate fluctuations in PFAS concentrations (ng L^−1^) across five sampling events, highlighting consistently elevated levels at Point 1 and moderate temporal variability at several downstream locations.

Temporal variation observed during the sampling campaign (Figure 5) indicates that PFOS remained consistently elevated at Point 1 throughout the monitoring period, with a noticeable increase during the final sampling event. At downstream sites, temporal variability was generally less pronounced but remained observable at Points 3, 4, and 6, where PFOS and PFOA concentrations fluctuated moderately between sampling dates. These variations likely reflect short-term hydrological dynamics influencing contaminant transport and mixing within the river system.

Overall, the spatial distribution of PFAS concentrations (Figure 4) highlights the dominant influence of localized contamination sources, while the temporal patterns observed across sampling sites (Figure 5) demonstrate dynamic variations in PFAS transport within the river network.

## 3. Discussion

### 3.1. Sources of Polymer-Related Contaminants from Landfill Leachate

The suspect screening approach provided evidence for the occurrence of methacrylate-based monomers, including diethylene glycol dimethacrylate (DEGDMA), triethylene glycol dimethacrylate (TEGDMA), and structurally related dimethacrylates, in landfill leachate and adjacent aquatic environments. These compounds share α,β-unsaturated carbonyl structures and glycol-derived backbones characteristic of polymerizable intermediates used in industrial resin formulations [29,30,31]. All were consistently detected at sampling points influenced by landfill inputs (Points 1, 3, 4, and 5) and were absent at the upstream reference site (Point 2), supporting the interpretation that the Brånåsen landfill is a likely local contributor of contaminants linked to polymeric materials. However, source attribution should be interpreted cautiously, because the monitored river reach may also be influenced by additional inputs, including urban runoff, road runoff, wastewater-related discharges, and diffuse industrial or commercial activities in the surrounding catchment. Therefore, while the spatial distribution of these compounds is consistent with a landfill-leachate contribution, the available dataset does not allow landfill inputs to be isolated as the sole source, in agreement with previous studies highlighting landfill leachate as a significant source of emerging contaminants to aquatic systems [32,33].

The co-occurrence of multiple dimethacrylate species, together with the detection of chromatographic peaks sharing identical nominal masses, supports the release of complex mixtures of residual monomers, structural isomers, and/or transformation products from polymeric materials disposed of at the site. Nevertheless, similar compounds may also be released from polymer-containing materials present in urban and industrial runoff, and these alternative diffuse sources cannot be excluded based on the present dataset.

DEGDMA, characterized by a relatively low log Kow (0.81), exhibits high mobility in the aqueous phase and limited sorption to organic matter [29], facilitating its transport via landfill leachate into receiving waters. In contrast, TEGDMA (also referred to as TGM3 in commercial formulations) is a low molecular weight dimethacrylate diluent/co-monomer widely used in resin-based composite materials, particularly dental composites. Although direct information on its occurrence, persistence, and degradation in environmental matrices remains limited, its environmental relevance is supported by evidence that TEGDMA can leach from cured resin matrices into aqueous and aqueous-organic media. This behavior has been attributed to its relatively low molecular weight and hydrophilic character, which favor mobility within and elution from the polymer matrix [34,35]. Together, these observations suggest that DEGDMA may be readily transported in the dissolved phase, whereas TEGDMA should be regarded as a leachable resin-based composite monomer with potential environmental relevance, particularly where methacrylate-containing polymeric wastes are exposed to water or landfill leachate.

These findings highlight landfills as potential ongoing contributors of polymer-derived contaminants, as plastics and resin-based materials can degrade over time and release residual monomers, cross-linkers, and additives into surrounding aquatic systems, contributing to complex mixtures of contaminants that are often not included in routine monitoring programs [14,36,37]. From an environmental perspective, the detection of dimethacrylate-related candidates is relevant because these compounds may behave as residual monomers or polymer-associated additives released from resin-based, adhesive, coating, and plastic materials. Their potential concern is related not only to direct toxicity, but also to their persistence, mobility, and occurrence as complex mixtures of parent compounds, structurally related analogues, and possible transformation products. In addition, their limited inclusion in routine monitoring and regulatory frameworks means that their occurrence in landfill-impacted aquatic systems may be underreported compared with better-known contaminant classes such as PFAS. The identification of DEGDMA, TEGDMA, and related compounds therefore underscores both the environmental relevance of methacrylate monomers and the value of suspect screening using high resolution mass spectrometry for detecting overlooked contaminants associated with polymer-containing waste streams, while also pointing to the need for further research on their fate, transformation, toxicity, persistence, and occurrence in other environmental compartments such as sediments and biota.

### 3.2. Occurrence and Distribution of PFAS in the River System

In addition to polymer-derived compounds, the detection of several PFAS along the monitored section of the Nitelva River indicates the presence of persistent fluorinated contaminants within the local aquatic environment. The elevated PFOS and PFOA concentrations observed at the landfill-associated tributary are consistent with a possible landfill-leachate contribution to the river system. Landfills are widely recognized as secondary reservoirs of PFAS due to the accumulation of fluorinated compounds originating from consumer products, industrial materials, and firefighting foams disposed of in municipal waste streams [38,39,40]. Recent investigations continue to confirm the role of landfill sites as long-term sources of PFAS contamination. Studies have shown that PFAS released from landfill leachate can migrate into nearby surface waters and create persistent contamination plumes in aquatic ecosystems [41]. Reviews of PFAS occurrence in landfill leachate similarly highlight waste disposal sites as major pathways for PFAS release into the environment, due to the accumulation of fluorinated substances in municipal and industrial waste [40,42].

The elevated PFAS concentrations observed at the landfill tributary in the present study therefore support previous findings that landfill sites can act as long-term reservoirs of PFAS contamination in freshwater environments [43]. The difference in PFAS concentrations between the landfill-associated tributary and the upstream reference site suggests lower PFAS levels in the main river upstream of the landfill influence. Downstream, additional potential inputs may be associated with surrounding urban, industrial, and infrastructural areas, including the Kjeller military airport, Dynea AS, and the nearby urban area of Lillestrøm; however, their individual contributions cannot be confirmed or quantified from the present dataset. The pronounced concentration gradient suggests the possible influence of localized inputs, although the relative contribution of landfill leachate, urban runoff, industrial inputs, and other point or diffuse sources cannot be quantitatively resolved with the available data. In contrast, atmospheric deposition may represent an additional diffuse input pathway, although its relative importance in this system cannot be directly assessed from the present dataset, despite the known potential for long-range atmospheric transport of PFAS [44,45].

### 3.3. Transport and Dilution Processes in the Nitelva River

Although the highest PFAS concentrations were detected at the landfill-impacted tributary, measurable concentrations persisted throughout the downstream river reach, demonstrating the high mobility and environmental persistence of these compounds in aquatic systems. The progressive decrease in PFAS concentrations observed along downstream sampling sites suggests that dilution and hydrological mixing within the main river channel may have contributed to the observed concentration gradient. However, because site-specific hydrological data, such as river discharge, tributary flow rates, and time-resolved flow measurements, were not available, this interpretation should be considered qualitative rather than quantitative. Therefore, the downstream decrease in PFAS concentrations should be interpreted as apparent attenuation along the monitored reach rather than as a direct quantification of dilution or transport processes.

The persistence of PFAS downstream from the contamination source is consistent with the well-documented chemical stability of these compounds. PFAS are characterized by extremely strong carbon–fluorine bonds that confer resistance to biological, chemical, and photolytic degradation, allowing them to persist in aquatic environments and undergo long-distance transport [46,47,48]. Recent global assessments further indicate that PFAS contamination is widespread in freshwater systems, with a large proportion of surface waters worldwide containing detectable concentrations of these substances [49]. In addition, PFAS contamination has been proposed to exceed newly defined planetary boundaries for chemical pollution, highlighting the global scale of this environmental issue [50], including snowmelt-driven runoff and episodic precipitation events. These conditions may influence contaminant transport, mixing dynamics, and persistence in river systems.

It should also be noted that precipitation events occurred during the sampling week, which likely caused additional dilution of the aqueous samples and may therefore have resulted in a partial underestimation of the measured contamination levels. The moderate temporal fluctuations observed in PFAS concentrations at several downstream sampling points may reflect short-term hydrological processes influencing contaminant transport, including variations in river discharge and mixing between tributaries and the main river channel. However, in the absence of concurrent hydrological measurements, these processes cannot be resolved quantitatively and should be interpreted as plausible explanations rather than confirmed transport mechanisms.

### 3.4. Environmental Implications and Monitoring Considerations

The combined detection of PFAS and polymer-related additives highlights the complex mixture of emerging contaminants that may originate from legacy landfill sites. At the same time, the possible contribution of other sources, including urban runoff, wastewater-related inputs, road runoff, and nearby industrial activities, should be considered when interpreting contaminant patterns in complex river catchments.

Although PFAS concentrations decreased downstream of the contamination source, detectable levels persisted throughout the monitored river reach, indicating that contaminants released from landfill leachate can be transported over considerable distances within freshwater systems. This interpretation is consistent with the observed spatial concentration patterns but remains qualitative because hydrological data were not available to constrain dilution and transport processes. The environmental persistence and potential toxicity of PFAS raise concerns regarding long-term ecological impacts, including bioaccumulation in aquatic organisms and potential human exposure pathways through water use and recreational activities [46]. Increasing regulatory attention toward PFAS contamination reflects growing recognition of the need to better understand their sources, environmental behavior, and long-term impacts on ecosystems [51].

The tentative identification of polymer-derived additives further demonstrates that landfill sites may release a broader spectrum of emerging contaminants beyond well-known pollutant classes. These compounds are environmentally relevant because polymer-derived additives and residual monomers may persist in aquatic systems, partition between dissolved and particulate phases, and occur as complex mixtures of structurally related compounds that are not routinely monitored. Although compound-specific ecotoxicological data for many dimethacrylate-related substances in natural waters remain limited, their occurrence in landfill-impacted waters indicates a potential exposure pathway that warrants further investigation. From a regulatory perspective, these findings also highlight a gap between the increasing attention given to well-established contaminants such as PFAS and the more limited monitoring of polymer-associated chemicals released from waste materials. These findings emphasize the importance of combining suspect screening approaches with targeted analytical methods when investigating complex contamination sources. Suspect screening enables the detection of previously unmonitored compounds associated with specific industrial activities, while targeted quantification provides reliable concentration data for well-known contaminants such as PFAS. The integration of these complementary analytical approaches therefore offers a more comprehensive understanding of contaminant occurrence, sources, and environmental behavior. Additionally, given that PFAS exhibit high chemical stability due to strong C–F bonds—supporting persistence and long-distance transport in aquatic systems—and that polymer-derived additives such as TEGDMA may partition between dissolved and particulate phases (including sediments), future monitoring should explicitly include fluvial sediments to evaluate potential accumulation, persistence, and longer-term secondary release, and to better constrain contaminant fate in landfill-impacted reaches.

Overall, the results of this study support the interpretation that legacy landfill sites may act as persistent contributors of both fluorinated contaminants and polymer-derived chemicals in freshwater environments. However, the findings also underline the importance of considering alternative diffuse and point sources, including urban runoff, wastewater-related inputs, road runoff, and industrial activities, when attributing contaminant sources in complex river systems. Continued monitoring of landfill-impacted waters, together with improved characterization of landfill leachate composition, fluvial sediments, hydrological conditions, and polymer-derived additive toxicity, persistence, and regulatory relevance, will be essential for assessing long-term environmental risks and supporting the development of effective water quality management strategies.

## 4. Materials and Methods

### 4.1. Area of Study

The area of study is the Skedsmo municipality, located in Akershus County in Norway, approximately 20 km northeast of Oslo. This subarctic region presents a diverse landscape shaped by both natural processes and anthropogenic pressures. Over the past decades, the area has experienced significant urban and industrial development, leading to complex environmental pressures on local ecosystems. Skedsmo is characterized by a mix of residential zones, commercial activities and important industrial installations, that have historically influenced the quality of the surrounding environment. Despite the urban expansion, rural landscapes persist in parts of the municipality, where agricultural fields and natural areas are still present. The territory is also traversed by several watercourses that converge into the Nitelva River: it emphasized the strong interaction between land use and the aquatic environment.

A major anthropogenic hotspot in the area is the Brånåsen waste landfill, established in 1970 and decommissioned around 1990, before modern containment standards were implemented [52]. For years, the site served as a disposal area for industrial waste, including contributions from Dynea AS (Norway), a chemical company specializing in adhesives and resins. Residential development in the surrounding area began in the 1990s and by the early 2000s, housing expanded onto the landfill itself. In 2012, the municipality of Skedsmo reported hazardous gas emissions inside some residential houses, prompting the redemption of affected properties and triggering ongoing investigations into appropriate mitigation measures [53]. Due to the lack of adequate containment barriers and leachate treatment systems, the Brånåsen landfill has long been considered a significant source of contamination through leachate release.

### 4.2. Sampling Collection

The samples were taken for one week in consecutive days. The weather conditions were cloudy, with medium–low rain showers. No abnormal rains or overflows were registered before the sampling collection. Water samples were collected from seven distinct locations along the Nitelva river, as shown in Figure 6, in order to capture spatial variability in contaminant distribution and potential inputs from anthropogenic sources. The selection of the points was based on both hydrological relevance and proximity to known or suspected contamination sources. The sampling collection was performed in the points described at Figure 6.

**Figure 7:**
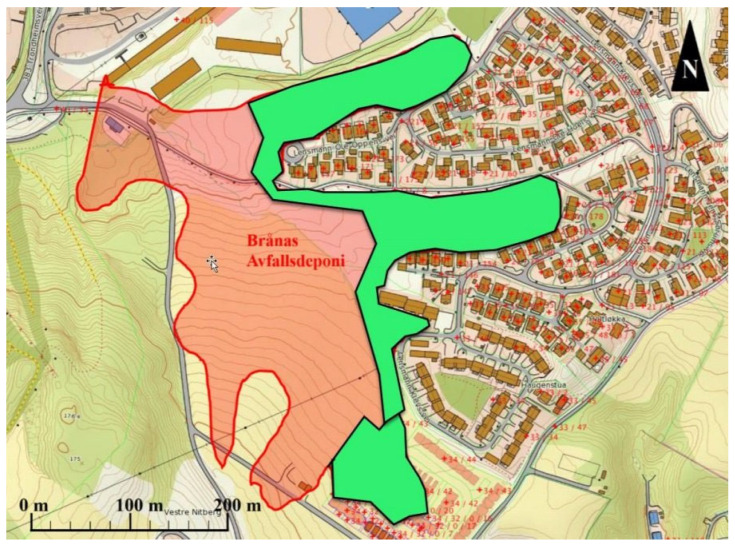
Brånåsen landfill area and sampling point 1 (raw landfill leachate, point where urban system collects the landfill leachate).

The seven sampling points were established along the Nitelva River to characterize spatial variations in water quality and to assess the influence of both point and diffuse pollution sources. Point 1 corresponds to an urban rainwater collector located beside the Brånåsen landfill, where landfill leachate infiltration has been identified (Figure 7). This collector receives leachate conveyed by a small tributary draining the closed landfill and represents the only accessible water compartment directly connected to the site prior to discharge into the Nitelva. As the confluence of this tributary with the river occurs downstream of Point 2, the latter was selected immediately upstream of the inflow to provide a baseline reference for river water quality before any direct influence from the landfill effluent. Points 3 and 4 were located further downstream within a more urbanized river reach, near bridges and major road infrastructure, including areas adjacent to Skjetten and beneath the Trondheimsveien highway. These sites were chosen to evaluate the potential impacts of urban runoff, traffic-related emissions, and surrounding residential activities. Point 5 was situated further southeast, downstream of the urban-influenced sites, to assess the dilution, transport, and persistence of contaminants introduced upstream. This location represents an intermediate stretch of the river before it enters a wider, semi-natural basin. Point 6 was established within the Nebbusvollen Friluftsbasseng recreational area, a site of particular relevance for evaluating water quality under conditions of potential direct human exposure, especially during the warmer months. Finally, Point 7 was positioned further downstream near the town of Lillestrøm, prior to the river crossing major infrastructural zones and merging into broader water systems. This site was selected to capture the cumulative effects of upstream inputs, integrating contributions from both point sources, such as landfill leachate, and diffuse sources associated with urban and semi-urban land use.

Two liters of water samples were systematically collected from the seven predetermined locations along the river course and stored in sterile plastic bottles (Astik’s) specifically designed for microbiological and chemical analysis. Bottles were prewashed (with the same sampling water), filled to the top to avoid oxygen gap, and transported to the laboratory right after the sampling. For the sample collection, a manual pump system connected to a water hose with 6 m of distance was used. Following sampling, the bottles were promptly refrigerated at 4 °C to preserve the integrity of the samples prior to pre-treatment and analysis.

### 4.3. Sample Pretreatment

#### 4.3.1. SPE for Acrylamide-Type Suspect Screening

For the suspect screening analysis, water samples were filtered prior to solid-phase extraction (SPE) to remove suspended particulate matter. Filtration was performed using a vacuum-assisted system with hydrophilic cellulose acetate membranes (0.45 μm pore size, 47 mm diameter). The filtrates were collected and then employed in the SPE process to isolate polar analytes (such as the acrylamide-type substances and PFOA) from water samples using ISOLUTE^®^ ENV+ cartridges (Biotage^®^, Uppsala, Sweden). They contain a highly retentive non-polar sorbent specifically designed to retain polar compounds that are typically not captured by conventional C18 or C8 phases. Prior to loading, each water sample was acidified to a pH of 2 by adding hydrochloric acid (HCl 37%). The SPE cartridges were pre-conditioned sequentially with 10 mL of methanol (MeOH), followed by 5 mL of 0.1 M HCl, to activate and equilibrate the sorbent material. A total of 500 mL of acidified sample was then passed through each cartridge under vacuum at a controlled flow rate, using a SPE Tube Vacuum Manifold (Phenomenex^®^, Torrance, CA, USA) (12–24 samples). This system enabled parallel processing of multiple samples under consistent conditions. After sample loading, a 2 mL wash with Milli-Q water (Millipore^®^, Darmstad, Germany) was applied to remove matrix interferences without eluting the target analytes.

To ensure the complete removal of residual water, the cartridges were dried under a gentle stream of nitrogen (N_2_) for approximately 30 min. Elution was subsequently performed with 1.5 mL of dry acetonitrile (dried ACN) and the analytes were collected in glass tubes, which offer chemical compatibility and minimize analyte loss due to adsorption or evaporation. The eluates were then transferred into glass vials equipped with septum caps to reduce volatilization. Each vial was further sealed with parafilm and stored at 4 °C in a refrigerator until analysis.

#### 4.3.2. SPE for PFAS Quantification

Solid-phase extraction (SPE) was employed to isolate per- and polyfluoroalkyl substances (PFAS) from water samples using Bakerbond Octadecyl (C18) cartridges (Avantor^®^ Center Valley, PA, USA) containing a non-polar reversed-phase sorbent suitable for retaining hydrophobic PFAS compounds. Prior to extraction, 50 mL of each water sample was centrifuged at 5000 rpm for 3 min using a Heraeus Megafuge 16 (Thermo Scientific^®^, Osterode am Harz, Germany) to remove suspended particulate matter and minimize matrix interference. The SPE cartridges were conditioned with methanol to activate the sorbent and enhance analyte retention. The clarified supernatant was subsequently loaded onto the cartridges under controlled vacuum conditions, allowing hydrophobic PFAS to partition onto the C18 stationary phase while the aqueous matrix passed through the sorbent. Following sample loading, the cartridges were air-dried under vacuum for approximately 5 min to remove residual water. Retained analytes were then eluted with methanol, yielding 5 mL of methanolic extract from the initial 50 mL water sample, corresponding to a tenfold concentration factor. The extracts were collected in polypropylene tubes to minimize potential contamination and stored at 4 °C until instrumental analysis.

### 4.4. Analytical Methods and Data Processing for Suspect Screening

An analytical workflow was developed for the detection of acrylamide-type and structurally related α,β-unsaturated vinyl monomers and perfluorinated organic substances in environmental samples. The method was designed to ensure high selectivity for polar, polymerizable compounds characterized by unsaturated carbonyl functionalities.

To cover the maximal number of substances (from small-to-medium and volatile-to-less volatile monomers), the suspect screening was performed using two different instrumentations, gas chromatography coupled to mass spectrometry (GC-MS/MS) and liquid chromatography coupled to high resolution mass spectrometry (UHPLC–HRMS). UHPLC–HRMS–based suspect screening was applied to support the tentative identification of PFAS candidates in the samples, leveraging the broad analytical coverage of LC–HRMS for short- and long-chain PFAS, including non-volatile and semi-volatile species. Whereas methylacrylamide-like components were more confidently identified using GC–MS/MS, which provided more diagnostic information for this compound class.

For the screening analysis method by GC-MS/MS, we used a Thermo Scientific TraceTM 1310 gas chromatograph (Thermo Fischer Scientific, Waltham, MA, USA) equipped with a Restek Rtx^®^-5MS column (30 m × 0.25 mm × 0.25 μm) and coupled with a triple quadrupole mass spectrometer Thermo Scientific TSQ 8000 (Thermo Fischer Scientific, Waltham, MA, USA). The temperature program of the oven was as follows: initial temperature 50 °C kept for 3 min, followed by a ramp of 20 °C/min to 110 °C, and another ramp of 15 °C/min to 290 °C, and finally 7 min at 290 °C. Helium with a purity of 99.999% (Praxair Norway AS, 0663 Oslo, Norway) was used as carrier gas at a constant flow of 1 mL/min. The temperature of the injector was 250 °C and the temperatures of the ion transfer line and ion source were 290 °C and 320 °C, respectively. The injector was operated in split-less mode for 2 min returning to split mode after this time. The mass spectrometer (MS) was operated in electron ionization mode (+70 eV) and full scan mode was used to monitor suspected molecules within the range of 75–600 Da. The operation conditions of the MS were standard for screening purposes.

For the screening analysis method by UHPLC–HRMS, the chromatographic separation was performed on an Acquity BEH C18 column (150 × 2.1 mm, 1.7 μm particle size, Waters Corporation^®^ Milford, MA, USA) using a Dionex Ultimate 3000 UHPLC system (Thermo Scientific, Germering, Germany). The mobile phase consisted of water with 0.1% formic acid (A) and methanol (B). A gradient program was applied starting at 95% A, decreasing to 0% A within 8 min, followed by column re-equilibration to initial conditions. The flow rate was 0.5 mL min^−1^, and the injection volume was 10 µL. Mass spectrometric analysis was carried out using a Q Exactive™ Orbitrap mass spectrometer (Thermo Fisher Scientific^®^ GmbH, Bremen, Germany) equipped with a heated electrospray ionization (HESI) source operating in both positive and negative ion mode. Full MS data were acquired at 70,000 resolution (FWHM at *m*/*z* 200) over the *m/z* range 50–750. Data-dependent MS^2^ (dd-MS^2^) experiments were performed at 35,000 resolution using higher-energy collisional dissociation (HCD) with normalized collision energy (NCE) set to 35%. To correct for HESI-related matrix effects during sample injection (signal suppression/enhancement caused by background), tripropylamine ([M-H]^+^ 144.2) was added as an internal standard immediately prior to injection (added to reach 10 µg L^−1^ in the sample extract) and used to normalize instrument response across injections.

Candidate features were retained only when chromatographic peaks showed consistent peak shape and were absent or negligible in procedural blanks. For UHPLC–HRMS data, candidates were further filtered using precursor-ion mass errors within ±5 ppm, isotopic-pattern agreement with the theoretical distribution predicted for the proposed elemental formula, and dd-MS^2^ evidence based on diagnostic product ions, characteristic neutral losses, spectral-library matches, and chemically plausible fragmentation pathways. For GC–MS/MS data, tentative assignments were supported by diagnostic fragmentation patterns and available spectral-library matches. Candidates showing inconsistent spectral evidence or ambiguous structural assignment were either rejected or assigned lower identification confidence, as described in the Results Section according to the Schymanski framework.

### 4.5. Analytical Quantification Method and Data Processing for PFAS

Based on the outcomes of the suspect screening, a targeted quantitative analysis was subsequently performed for selected PFAS compounds. Water samples were collected along the Nitelva River and subjected to solid-phase extraction (SPE) to isolate per- and polyfluoroalkyl substances (PFAS), yielding methanolic extracts that were subsequently analyzed by liquid chromatography–tandem mass spectrometry (LC–MS/MS). Analyses were performed using an ExionLC™ system coupled to a SCIEX QTRAP^®^ 6500+ triple quadrupole mass spectrometer (SCIEX, Framingham, MA, USA). Chromatographic separation was achieved on a reversed-phase C18 column (100 × 2.1 mm, 1.6 µm particle size, 100 Å pore size), and 100 µL of each methanolic extract was injected for analysis. The mass spectrometer was operated in negative electrospray ionization (ESI–) mode, and detection was carried out using multiple reaction monitoring (MRM). Optimized transitions and MS/MS parameters for PFAS quantification are provided in Table S1 of the Supplementary Materials. Quantification was performed using a seven-point external calibration curve prepared in methanol over a concentration range of 5–200 ng/L with linear regression and 1/x weighting. PFAS analytical standards were obtained from Wellington Laboratories (Guelph, ON, Canada), Ultra Scientific (North Kingstown, RI, USA), and Agilent Technologies (Santa Clara, CA, USA). Isotope-labeled internal standards obtained from Wellington Laboratories (MPFAC-C-ES mixture, Guelph, ON, Canada), including ^13^C_4_-PFBA, ^13^C_2_-PFDA, ^13^C_2_-PFDoA, ^13^C_2_-PFHxA, ^13^C_5_-PFNA, ^13^C_4_-PFOA, ^13^C_2_-PFUnA, ^18^O_2_-PFHxS, and ^13^C_4_-PFOS, were included for most analytes to monitor instrumental performance and potential matrix effects, although analyte concentrations were not recovery-corrected. Additional details regarding analytical standards and isotope-labeled internal standards are provided in Tables S2 and S3 of the Supplementary Materials, respectively. The instrumental limit of quantification (LOQ) was approximately 10 ng/L for most PFAS compounds, while PFBS exhibited a higher LOQ of 20 ng/L due to background environmental contamination. Limits of detection (LOD) were not assessed, as the study focused on quantitative reliability at the LOQ level. Quality assurance and quality control procedures included laboratory blanks and continuing calibration verification standards (50 ng/L) analyzed every 20 samples, and calibration curves were accepted when the coefficient of determination (R^2^) exceeded 0.98. Because the injected extract represented a tenfold pre-concentration of the original water sample (50 mL reduced to 5 mL during SPE), instrumental concentrations were corrected by dividing by 10 to report final PFAS concentrations in the original water matrix (ng/L). Data acquisition and processing were conducted using the instrument manufacturer’s software, with manual review of chromatographic integration to ensure consistency and analytical reliability.

## 5. Conclusions

This work provides the first combined evidence of cross-linker used in polymer materials and multiple PFAS co-occurring in surface waters influenced by the Brånåsen legacy landfill. By integrating GC-MS/MS, UHPLC-HRMS suspect screening with targeted LC–MS/MS across an upstream–downstream transect, we localize acrylamide-type compounds to the landfill-associated tributary and resolve marked PFAS gradients in the Nitelva. The approach operationalizes a practical blueprint for legacy-landfill surveillance that simultaneously addresses polymer additives and PFAS, thereby extending current monitoring beyond conventional PFAS-only assessments. The results indicate that the landfill represents a localized source of contamination to the surrounding aquatic environment. Suspect screening identified diethylene glycol dimethacrylate (DEGDMA) and triethylene glycol dimethacrylate (TEGDMA) at the landfill-impacted site, highlighting the release of adhesive-related compounds associated with historical industrial waste disposal. In parallel, targeted analysis revealed elevated PFAS concentrations at the tributary receiving landfill leachate, with decreasing concentrations observed downstream due to dilution and hydrological mixing within the river system. Despite this attenuation, detectable PFAS levels persisted along the monitored river reach, reflecting their high persistence and mobility in freshwater environments. These findings highlight the role of legacy landfill sites as potential sources of both fluorinated contaminants and polymer-related additives in freshwater environments. However, other sources of PFAS contamination may play a significant role such as the military airport beside the Nitelva river and after the landfill site.

As the EU implements the recast Urban Wastewater Treatment Directive (UWWTD, (EU) 2024/3019) and revises the Water Framework and Environmental Quality Standards Directives, the regulatory landscape is shifting toward stricter control of micropollutants, expanded monitoring, and enhanced accountability through Extended Producer Responsibility (EPR). These developments directly relate to the contaminants identified in this work. The UWWTD introduces mandatory quaternary treatment for micropollutant removal in large wastewater treatment plants and requires improved monitoring of PFAS, microplastics, and other emerging contaminants. Although landfill leachate is not directly regulated under this directive, it frequently enters municipal networks or receiving water bodies where compliance obligations apply. This creates a strong incentive for municipalities to consider targeted pre-treatment of leachate to reduce PFAS and polymer-additive loads before reaching urban wastewater infrastructure. Simultaneously, the updated Water Framework Directive strengthens environmental quality standards and expands PFAS group assessments, encouraging more comprehensive surveillance strategies. Therefore, continued environmental monitoring of landfill-impacted waters is essential to better understand contaminant transport dynamics and to support the development of effective management and remediation strategies aimed at reducing contaminant inputs to aquatic ecosystems. Future monitoring should, as this work has presented, integrate suspect screening, targeted quantification, and hydrological context to support early detection and regulatory alignment. These efforts will help guide investments in treatment technologies and improve the protection of vulnerable freshwater environments.

## Figures and Tables

**Table 1:** Sampling points, location (coordinates), pH, temperature registered and collection dates.

			Sampling Collection Dates									
Point	Latitude	Longitude	8/10/24	8/10/24	9/10/24	9/10/24	10/10/24	10/10/24	11/10/24	11/10/24	14/10/24	14/10/24
			pH	T (°C)	pH	T (°C)	pH	T (°C)	pH	T (°C)	pH	T (°C)
1	59.984	11.021	6.0	8.1	6.0	8.0	7.5	8.0	7.0	8.0	5.8	7.0
2	59.981	11.001	5.5	8.0	6.0	8.0	5.0	8.5	4.7	7.0	5.8	5.0
3	59.980	11.012	6.0	8.0	6.0	8.0	5.8	9.0	5.0	7.0	5.5	7.0
4	59.979	11.012	5.5	9.0	6.0	9.0	5.5	9.0	5.0	7.0	5.8	7.0
5	59.976	11.012	6.0	7.0	6.0	8.0	5.0	9.0	4.5	6.0	4.7	5.0
6	59.962	11.028	6.0	8.0	6.0	8.0	4.7	9.0	4.5	7.0	4.7	6.0
7	59.947	11.061	6.0	8.0	6.0	8.0	4.7	9.5	8.0	7.0	6.0	7.0

**Table 2:** Summary of dimethacrylate-related features tentatively annotated during suspect screening, including retention time, detected sampling points, and nominal molecular mass. Peaks and corresponding MS spectra were extracted from the XICs at the reported retention times.

Compound	Retention Time (min)	Detected Sampling Points	Nominal Molecular Mass *m*/*z*
DEGDMA	13.19	Points: 1, 3, 4, 5	242
TEGDMA	15.21	Points: 1, 3, 4, 5	286
COMP286a	16.97	Points: 1, 3, 4, 5	286
COMP286b	18.54	Points: 1, 3, 4, 5	286
COMP286c	19.96	Points: 1, 4, 5	286

## Data Availability

The original contributions presented in this study are included in the article and Supplementary Materials. Further inquiries can be directed to the corresponding author.

## Supplementary Materials

The following supporting information can be downloaded at: https://www.mdpi.com/article/10.3390/molecules31111922/s1, Figure S1. Annotation of diethylenglycol dimethacrylate via NIST MS library; Figure S2. Tentative annotation of triethylenglycol di-methacrylate (TEGDMA) at point 1, 3, 4 and 5: Peak at 15.21 min with MS spectrum extracted from XIC at 15.21 min; Figure S3. Annotation of component triethylenglycol dimethacrylate (TEGDMA) via NIST MS library; Figure S4. Tentative annotation of the isomer COMP286a of triethylene glycol dimethacrylate (TEGDMA) at point 1, 3, 4 and 5. Peak at 16.97 min with MS spectrum at 16.97 min; Figure S5. Annotation of component COMP286a via NIST MS library; Figure S6. Extracted ion chromatogram for tentative annotation of the isomer COMP286b of triethylene glycol dimethacrylate (TEGDMA) at point 1, 4, and 5: Peak at 18.54 min with MS spectrum at 18.54 min; Figure S7. Annotation of component COMP286b via NIST MS library, Figure S8. Extracted ion chromatogram for tentative annotation of the isomer COMP286c of triethylene glycol dimethacrylate (TEGDMA) at point 1, 2, and 3: Peak at 19.96 min with MS spectrum at 19.96 min; Figure S9. Annotation of component COMP286c via NIST MS library; Table S1. MRM transitions and MS/MS parameters for targeted PFAS analysis by LC–MS/MS; Table S2. Analytical standards used for targeted PFAS quantification; Table S3. Isotope-labelled internal standards used for targeted PFAS quantification.

## Abbreviations

The following abbreviations are used in this manuscript:

DEGDMA	diethylene glycol dimethacrylate
TEGDMA	triethylene glycol dimethacrylate
PFAS	per- and polyfluoroalkyl substances
UHPLC–HRMS	Ultra-high-performance chromatography coupled to high resolution mass spectrometry
XIC	Extracted ion chromatograph
CECs	Contaminants of emerging concern
