# Supplementary material for: Suspect Screening of Polymer-Derived Additives in Accessible Water Compartments Nearby Brånåsen Landfill in Norway
Source: Molecules. 2026 Jun 2;31(11):1922. doi: 10.3390/molecules31111922 (PMC13258652; doi:10.3390/molecules31111922)
Supplement: Supplementary file 1 [file molecules-31-01922-s001.zip › molecules-4265227-supplementary.pdf]

Supplementary materials

Figures:

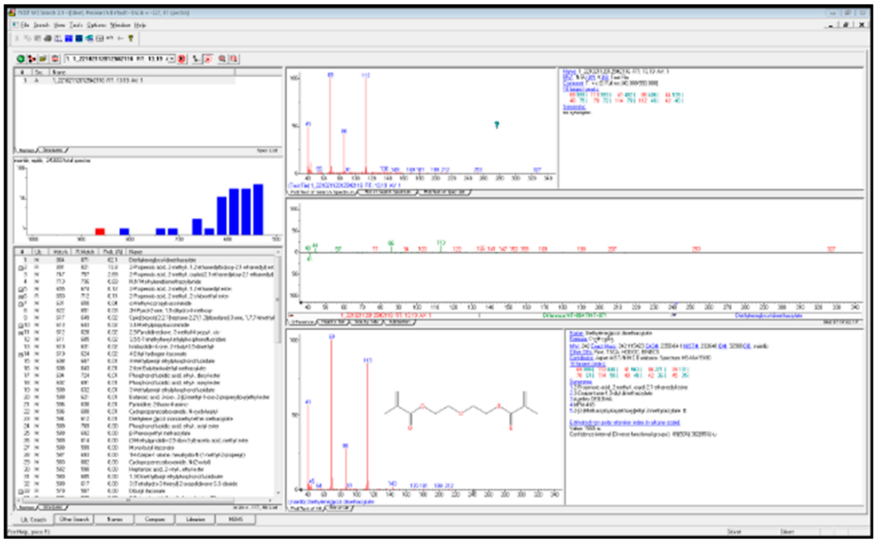

Figure S1. Annotation of diethylenglycol dimethacrylate via NIST MS library.

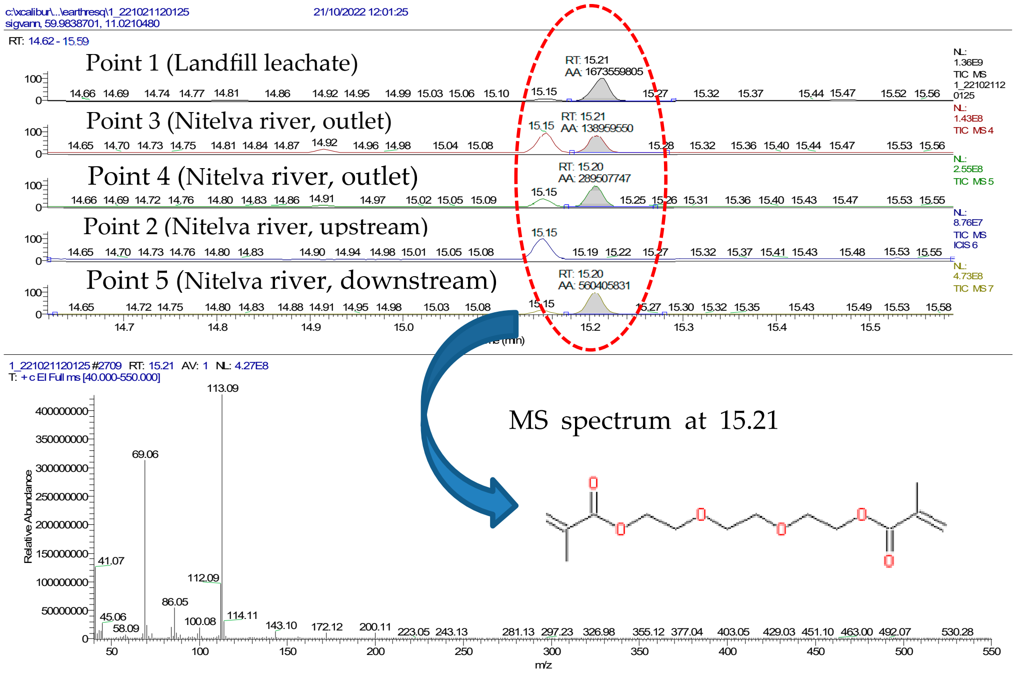

Figure S2. Tentative annotation of triethylenglycol di-methacrylate (TEGDMA) at point 1, 3,4 and 5: Peak at 15.21 min with MS spectrum extracted from XIC at 15.21 min.

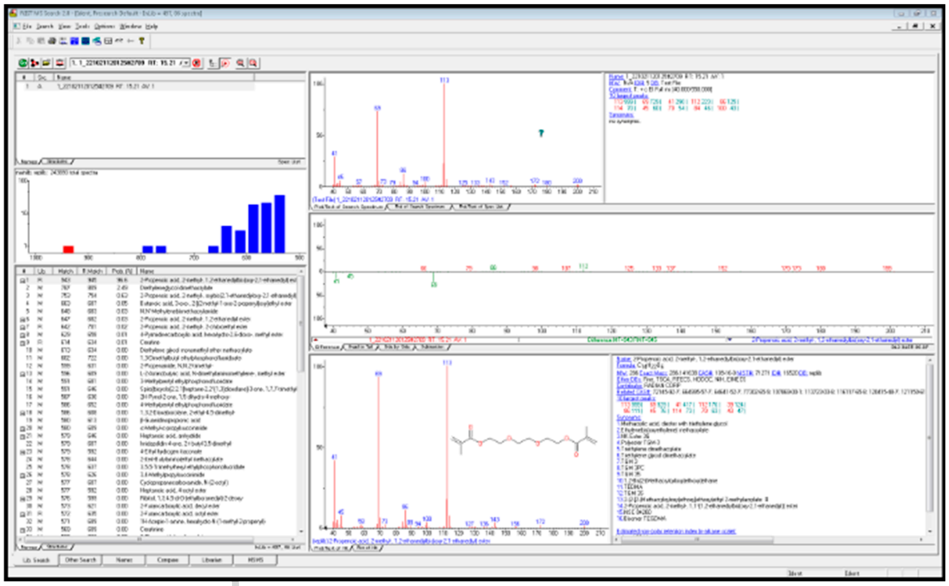

Figure S3. Annotation of component triethylenglycol dimethacrylate (TEGDMA) via NIST MS library.

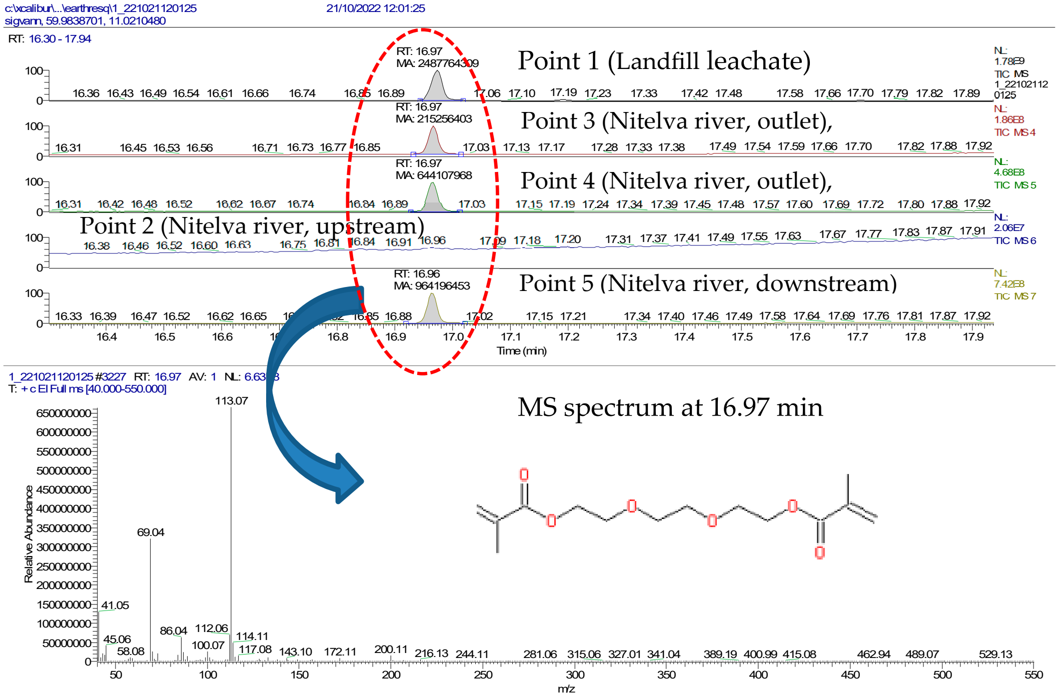

Figure S4. Tentative annotation of the isomer COMP286a of triethylene glycol dimethacrylate (TEGDMA) at point 1, 3,4 and 5. Peak at 16.97 min with MS spectrum at 16.97 min.

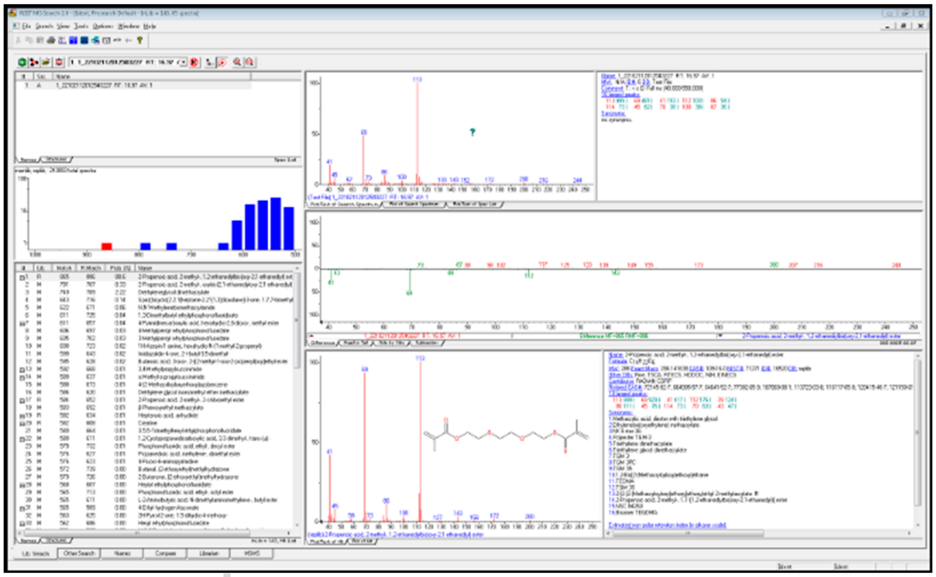

Figure S5. Annotation of component COMP286a via NIST MS library

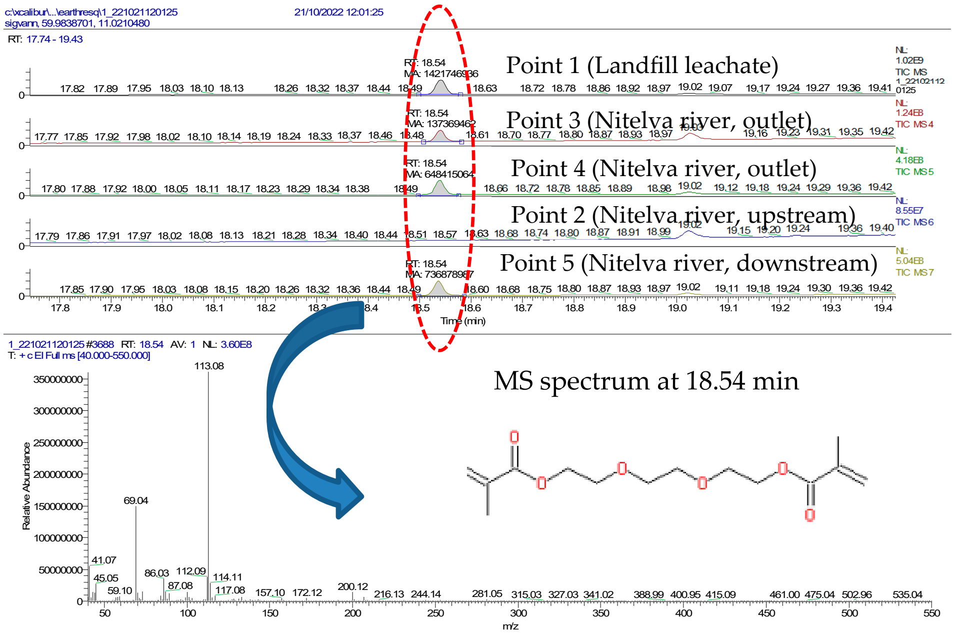

Figure S6. Extracted ion chromatogram for tentative annotation of the isomer COMP286b of triethylene glycol dimethacrylate (TEGDMA) at point 1, 4, and 5: Peak at 18,54 min with MS spectrum at 18.54 min.

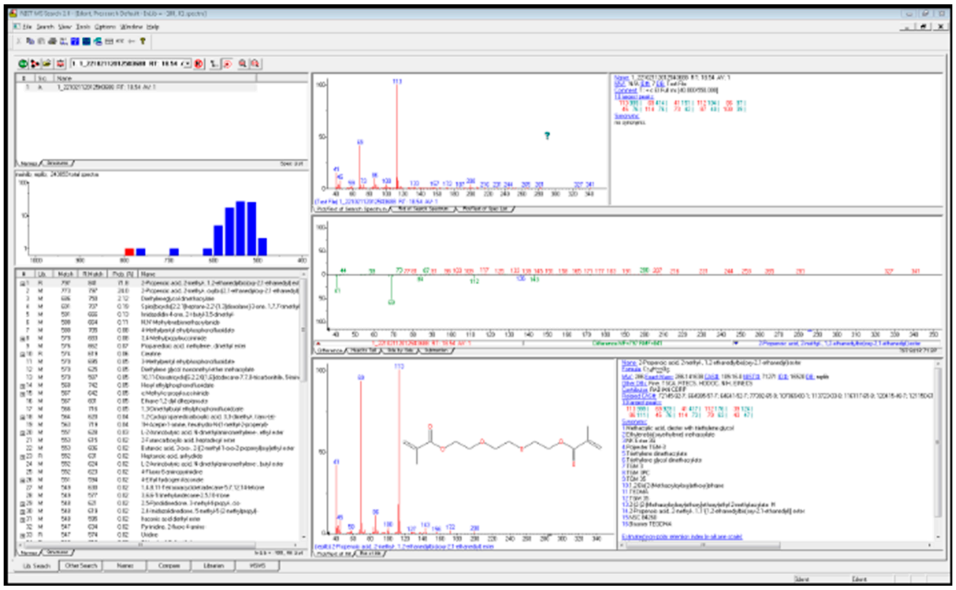

Figure S7. Annotation of component COMP286b via NIST MS library.

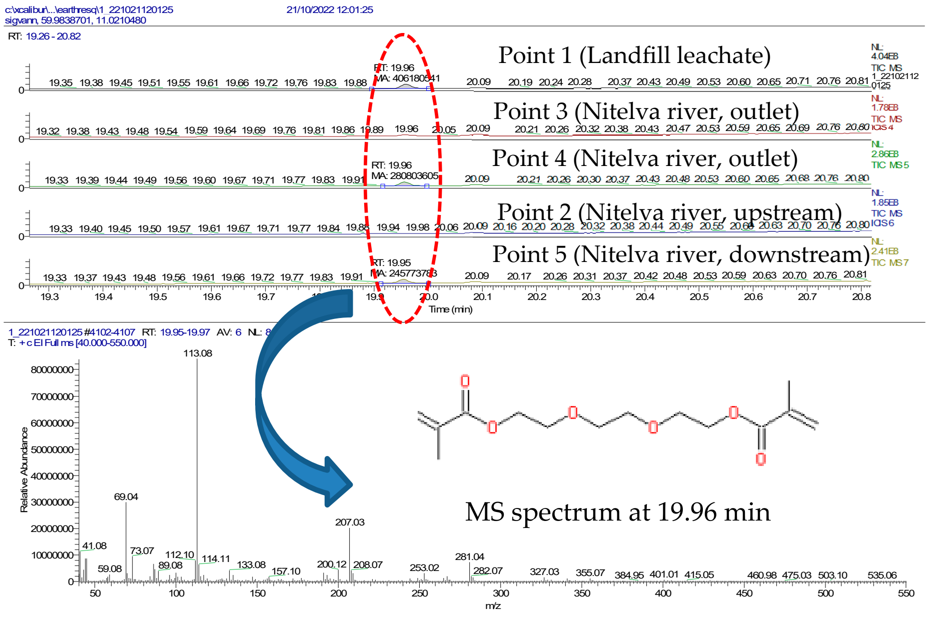

Figure S8. Extracted ion chromatogram for tentative annotation of the isomer COMP286c of triethylene glycol dimethacrylate (TEGDMA) at point 1, 2, and 3: Peak at 19,96 min with MS spectrum at 19.96 min.

IFE Public

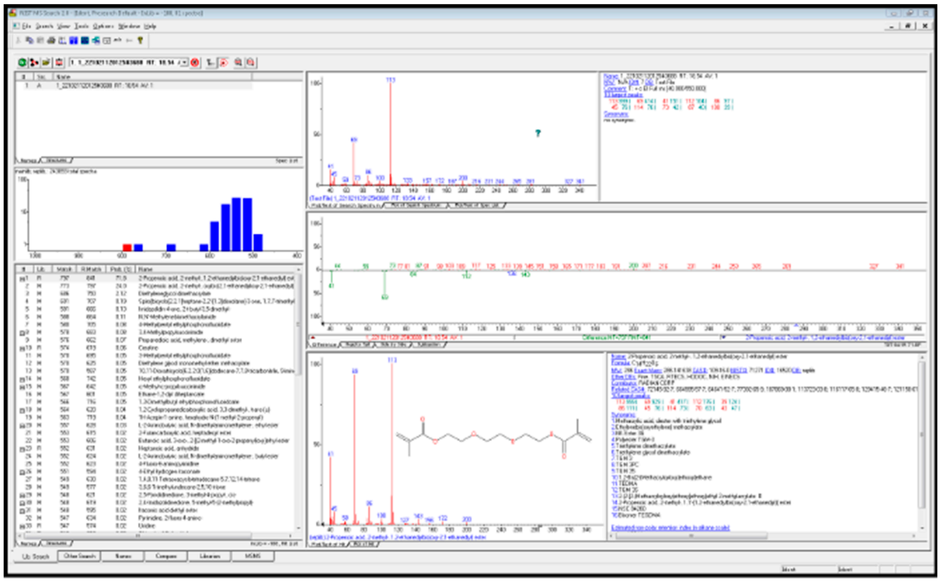

Figure S9. Annotation of component COMP286c via NIST MS library.

Tables:

Table S1. MRM transitions and MS/MS parameters for targeted PFAS analysis by LC–MS/MS

Analyte Transition	Q_1 (m/z)	Q_3 (m/z)	Q0D(Volts)	EP (Volts)	CE (Volts)	CXP (Volts)
M4-PFBA	217	172.000	-10.000	-10.000	-12.000	-10.000
PFBA 1	212.900	169.000	-10.000	-10.000	-12.000	-15.000
PFPeA 1	262.900	219.000	-10.000	-10.000	-12.000	-15.000
M5-PFPeA	268.000	223.000	-10.000	-10.000	-12.000	-15.000
PFBS 1	298.800	80.000	-80.000	-10.000	-58.000	-10.000
PFBS 2	298.800	99.000	-80.000	-10.000	-36.000	-10.000
M3-PFBS	302.000	80.000	-80.000	-10.000	-58.000	-12.000
M5-PFHxA	318.000	273.000	-10.000	-10.000	-16.000	-10.000
PFHxA 1	313.000	269.000	-10.000	-10.000	-16.000	-15.000
PFHxA 2	313.000	119.100	-10.000	-10.000	-30.000	-15.000
L-PFPeS 1	349.000	80.000	-80.000	-10.000	-86.000	-15.000
L-PFPeS 2	349.000	99.000	-80.000	-10.000	-38.000	-15.000
GenX 1	329.000	169.000	-10.000	-10.000	-18.000	-12.000
GenX 2	329.000	185.000	-10.000	-10.000	-32.000	-12.000
CC604 1	339.000	113.000	-20.000	-10.000	-35.000	-10.000

Analyte Transition	Q_1 (m/z)	Q_3 (m/z)	Q0D(Volts)	EP (Volts)	CE (Volts)	CXP (Volts)
CC604 2	339.000	85.000	-20.000	-10.000	-15.000	-10.000
M4-PFHpA	367.000	322.000	-20.000	-10.000	-12.000	-15.000
PFHpA 1	362.800	318.900	-20.000	-10.000	-12.000	-25.000
PFHpA 2	362.800	169.000	-20.000	-10.000	-22.000	-10.000
PFHxS 1	398.900	79.900	-80.000	-10.000	-95.000	-12.000
PFHxS 2	398.900	98.950	-80.000	-10.000	-80.000	-10.000
M3-PFHxS	402.000	80.000	-80.000	-10.000	-50.000	-12.000
ADONA 1	376.900	250.900	-20.000	-10.000	-15.000	-12.000
ADONA 2	376.900	84.900	-20.000	-10.000	-35.000	-12.000
PFOA 1	412.800	368.900	-30.000	-10.000	-13.000	-30.000
PFOA 2	412.800	168.700	-30.000	-10.000	-25.000	-10.000
M8-PFOA	421.000	376.000	-30.000	-10.000	-14.000	-15.000
L-PFHpS 1	449.000	80.000	-80.000	-10.000	-108.000	-15.000
L-PFHpS 2	449.000	99.000	-80.000	-10.000	-96.000	-15.000
M8-PFOS	507.000	80.000	-80.000	-10.000	-106.000	-12.000
PFOS 1	498.900	79.950	-80.000	-10.000	-106	-10.000
PFOS 2	498.800	98.950	-80.000	-10.000	-80.000	-10.000
PFNA 1	462.900	419.000	-20.000	-10.000	-16.000	-25.000
PFNA 2	462.900	219.000	-20.000	-10.000	-25.000	-25.000
M9-PFNA	472.000	427.000	-20.000	-10.000	-14.000	-15.000
L-PFNS 1	549.000	80.000	-90.000	-10.000	-134.000	-15.000
L-PFNS 2	549.000	99.000	-90.000	-10.000	-128.000	-15.000
PFDA 1	513.000	469.000	-30.000	-10.000	-15.000	-30.000
PFDA 2	513.000	269.000	-30.000	-10.000	-25.000	-30.000
M6-PFDA	519.000	474.000	-30.000	-10.000	-25.000	-15.000
PFDS 1	599.000	80.000	-80.000	-10.000	-130.000	-20.000
PFDS 2	599.000	99.000	-80.000	-10.000	-130.000	-35.000
PFUDA 1	563.000	269.000	-40.000	-10.000	-15.000	-18.000
PFUDA 2	563.000	219.000	-40.000	-10.000	-35.000	-18.000
M7-PFUnDA	570.000	525.000	-40.000	-10.000	-18.000	-15.000
PFUDS 1	648.900	80.000	-80.000	-10.000	-80.000	-12.000

Analyte Transition	Q_1 (m/z)	Q_3 (m/z)	Q0D(Volts)	EP (Volts)	CE (Volts)	CXP (Volts)
PFUDS 2	648.900	99.000	-80.000	-10.000	-99.000	-12.000
PFDoA 1	613.000	169.000	-40.000	-10.000	-13.000	-13.000
PFDoA 2	613.000	219.000	-40.000	-10.000	-12.000	-13.000
M2PFDoA	615.000	570.000	-30.000	-10.000	-13.000	-13.000
PFDoDS 1	699.000	80.000	-80.000	-10.000	-85.000	-12.000
PFDoDS 2	699.000	99.000	-80.000	-10.000	-80.000	-12.000
PFTrDA 1	663.000	169.000	-50.000	-10.000	-17.000	-15.000
PFTrDA 2	663.000	219.000	-50.000	-10.000	-40.000	-20.000
PFTrDS 1	748.900	80.000	-80.000	-10.000	-110.000	-12.000
PFTrDS 2	748.900	99.000	-80.000	-10.000	-80.000	-12.000
M2PFTeDA	715.000	670.000	-50.000	-10.000	-18.000	-15.000
PFTeDA 1	713.000	169.000	-50.000	-10.000	-42.000	-15.000
PFTeDA 2	713.000	219.000	-50.000	-10.000	-20.000	-15.000
PFHxDA 1	813.000	769.000	-40.000	-10.000	-40.000	-10.000
PFHxDA 2	813.000	169.000	-40.000	-10.000	-25.000	-15.000
HPFODA 1	913.000	869.000	-20.000	-10.000	-25.000	-15.000
HPFODA 2	913.000	169.000	-20.000	-10.000	-50.000	-20.000
6:2 FTS 1	427.000	407.000	-70.000	-10.000	-33.000	-9.000
6:2 FTS 2	427.000	81.000	-70.000	-10.000	-65.000	-4.000
M2 FTS	429.000	409.000	-70.000	-10.000	-35.000	-9.000

Table S2. Analytical standards used for targeted PFAS quantification

Compound	Supplier	Product code	Lot number
PFBA	Wellington Laboratories	PFAC-MXC	PFACMXC1222
PFPeA	Wellington Laboratories	PFAC-MXC	PFACMXC1222
PFBS	Wellington Laboratories	PFAC-MXC	PFACMXC1222
PFHxA	Wellington Laboratories	PFAC-MXC	PFACMXC1222
L-PFPeS	Wellington Laboratories	PFAC-MXC	PFACMXC1222

GenX	Wellington Laboratories	HFPO-DA	HFPODA1024
CC604	Ultra Scientific	CUS-00005916	6780719
PFHpA	Wellington Laboratories	PFAC-MXC	PFACMXC1222
PFHxS	Wellington Laboratories	PFAC-MXC	PFACMXC1222
ADONA	Wellington Laboratories	NaDONA	NaDONA1021
PFOA	Wellington Laboratories	PFAC-MXC	PFACMXC1222
L-PFHpS	Wellington Laboratories	PFAC-MXC	PFACMXC1222
PFOS	Wellington Laboratories	PFAC-MXC	PFACMXC1222
PFNA	Wellington Laboratories	PFAC-MXC	PFACMXC1222
L-PFNS	Wellington Laboratories	PFAC-MXC	PFACMXC1222
PFDA	Wellington Laboratories	PFAC-MXC	PFACMXC1222
PFDS	Wellington Laboratories	PFAC-MXC	PFACMXC1222
PFUdA	Wellington Laboratories	PFAC-MXC	PFACMXC1222
PFUDS	Wellington Laboratories	L-PFUdS	LPFUdS0122
PFDoA	Wellington Laboratories	PFAC-MXC	PFACMXC1222
PFDoS	Wellington Laboratories	PFAC-MXC	PFACMXC1222
PFTrDA	Wellington Laboratories	PFAC-MXC	PFACMXC1222
PFTrDS	Wellington Laboratories	L-PFTrDS	LPFTrDS0721
6:2FTS	Wellington Laboratories	6:2FTS	62FTS0722
MFS-N2	Agilent Technologies	CUS-00006479	6727219
MFS-M3	Agilent Technologies	CUS-00006480	6727220
MFS-N3	Agilent Technologies	CUS-00006481	6727221
MFS-M4	Agilent Technologies	CUS-00006482	6727222
MFS-N4	Agilent Technologies	CUS-00006483	6727223

MFS-N5	Agilent Technologies	CUS-00006484	6727224

Table S3. Isotope-labelled internal standards used for targeted PFAS quantification

Internal standard mixture	Supplier	Product code	Lot number	Composition
MPFAC-C-ES	Wellington Laboratories	MPFAC-C-ES	MPFACCES0623	MPFBA, M5PFPeA, M5PFHxA, M4PFHpA, M8PFOA, M9PFNA, M6PFDA, M7PFUdA, MPFDoA, M2PFTeDA, M3PFBS, M3PFHxS, and M8PFOS
